# Identification of circularRNAs and their targets in *Gossypium* under Verticillium wilt stress based on RNA-seq

**DOI:** 10.7717/peerj.4500

**Published:** 2018-03-16

**Authors:** Liuxin Xiang, Chaowei Cai, Jieru Cheng, Lu Wang, Chaofeng Wu, Yuzhen Shi, Jingzhi Luo, Lin He, Yushan Deng, Xiao Zhang, Youlu Yuan, Yingfan Cai

**Affiliations:** 1 State Key Laboratory of Cotton Biology, Henan Key Laboratory of Plant Stress Biology, School of Life Sciences, School of Computer and Information Engineering, Henan University, Kaifeng, Henan, China; 2 School of Bioinformatics, School of Software Engineering, Chongqing University of Posts and Telecommunications, Chongqing, China; 3 State Key Laboratory of Cotton Biology, Cotton Institute of the Chinese Academy of Agricultural Sciences, Key Laboratory of Cotton Genetic Improvement, Ministry of Agriculture, Anyang, Henan, China

**Keywords:** Circular RNA, Differential expression, Verticillium wilt, Cotton chromosome segment substitution lines, NBS family genes

## Abstract

Circular RNAs (circRNAs), a class of recently discovered non-coding RNAs, play a role in biological and developmental processes. A recent study showed that circRNAs exist in plants and play a role in their environmental stress responses. However, cotton circRNAs and their role in Verticillium wilt response have not been identified up to now. In this study, two CSSLs (chromosome segment substitution lines) of *G.barbadense* introgressed into *G. hirsutum*, CSSL-1 and CSSL-4 (a resistant line and a susceptible line to Verticillium wilt, respectively), were inoculated with *V. dahliae* for RNA-seq library construction and circRNA analysis. A total of 686 novel circRNAs were identified. CSSL-1 and CSSL-4 had similar numbers of circRNAs and shared many circRNAs in common. However, CSSL-4 differentially expressed approximately twice as many circRNAs as CSSL-1, and the differential expression levels of the common circRNAs were generally higher in CSSL-1 than in CSSL-4. Moreover, two C-RRI comparisons, C-RRI-vs-C-RRM and C-RRI-vs-C-RSI, possessed a large proportion (approximately 50%) of the commonly and differentially expressed circRNAs. These results indicate that the differentially expressed circRNAs may play roles in the Verticillium wilt response in cotton. A total of 280 differentially expressed circRNAs were identified. A Gene Ontology analysis showed that most of the ‘stimulus response’ term source genes were NBS family genes, of which most were the source genes from the differentially expressed circRNAs, indicating that NBS genes may play a role in Verticillium wilt resistance and might be regulated by circRNAs in the disease-resistance process in cotton.

## Introduction

In contrast with messenger RNA (mRNA), non-coding RNAs comprise the majority of the RNA world and include rRNA, lncRNA, snRNA, tRNA and miRNA, which function in various biological regulatory processes. In addition, circular RNA (circRNA) is a class of recently discovered non-coding RNA that features a circular structure (Hsu & Coca-Prados, 1979; Capel et al., 1993; Cocquerelle et al., 1993). CircRNAs are generated through head-to-tail back-splicing events (a junction between a downstream splice donor and an upstream splice acceptor) (Zhang et al., 2016; Gao et al., 2016). CircRNAs originate from exons, introns, and the intergenic region (Zhang et al., 2013; Jeck & Sharpless, 2014) and are generated from spliceosome-mediated precursor mRNA (pre-mRNA) splicing through a non-canonical splicing process in eukaryotes (Ashwal-Fluss et al., 2014; Chen, 2016). Recent study has shown that inhibition or slowing of canonical pre-mRNA processing events shifts the output of protein-coding genes toward circular RNAs (Liang et al., 2017). CircRNAs are observed in a diverse range of life forms, including archaea (Danan et al., 2012), mammals such as humans (Salzman et al., 2012), mice (Jiang et al., 2014; Pei et al., 2017), mosquitos (Gruner et al., 2016), sheep (Li et al., 2017), and plants such as rice (Lu et al., 2015; Ye et al., 2015), *Arabidopsis* (Ye et al., 2015; Sun et al., 2016; Pan et al., 2018; Dou et al., 2017), barley (Darbani, Noeparvar & Borg, 2016), and maize (Chen et al., 2018). This diversity suggests conserved biological functions and distinct properties. A recent study revealed that circRNAs play roles in biological and developmental processes. CircRNAs often show tissue-, cell- or developmental-stage-specific expression (Sun et al., 2016; Memczak et al., 2013; Salzman et al., 2013). CircRNAs are involved in the transcriptional and post-transcriptional regulation of gene expression (Memczak et al., 2013; Andreeva & Cooper, 2015; Li et al., 2015). CircRNAs can act as miRNA sponges to affect mRNA splicing and transcription (Wei et al., 2017; Gao et al., 2016; Hansen et al., 2013; Ebert, Neilson & Sharp, 2007; Franco-Zorrilla et al., 2007), and bind other ncRNAs or proteins to regulate the expression of other or even their parental genes (Han et al., 2017; Abdelmohsen et al., 2017). Moreover, circRNAs can serve as biomarkers of disease, such as Alzheimer’s disease and cancer (Sun et al., 2018; Lukiw, 2013; Li et al., 2015). Very recent studies have shown that circRNAs function as protein-coding sequences *in vitro* or *vivo* (Legnini et al., 2017; Pamudurti et al., 2017; Yang et al., 2017).

Although circRNAs are universally present across the eukaryotic tree of life (Wang et al., 2014), circRNAs in plants are reported with much less frequency than in mammalian research. Very recent reports have shown that circRNAs exist in plants, and circRNAs were comprehensively identified by an analysis of RNA-seq data in rice, *Arabidopsis*, barley, tomato, wheat, and soybean (Lu et al., 2015; Ye et al., 2015; Sun et al., 2016; Darbani, Noeparvar & Borg, 2016; Zuo et al., 2016; Wang et al., 2017; Zhao et al., 2017). CircRNAs in rice exhibit tissue-specific expression and regulate the expression of their parental genes (Lu et al., 2015). CircRNAs in barley function in response to the micronutrients iron and zinc (Darbani, Noeparvar & Borg, 2016). Sun et al. (2016) found that chloroplasts are an active area for circRNA generation, and complementary sequences exist in not only the intron regions but also the surrounding splice sites in *Arabidopsis*. Moreover, circRNAs have been suggested to play a role in the plant response to environmental stress. For example, the expression profiles of circRNAs have been reported for rice responding to Pi-starvation stress and in *Arabidopsis* responding to heat, low- and high-light stresses (Pan et al., 2018; Ye et al., 2015). The differential expression of circRNAs was reported in tomato fruits affected by chilling stress (Zuo et al., 2016). Wang et al. (2017) revealed a possible connection between the regulation of circRNAs and the expression of functional genes in wheat leaves associated with dehydration resistance. The mechanism of how circRNAs participate in the stress response is attributed to the fact that it exhibits alternative splicing circularization patterns and acts as a negative regulator of their parental genes (Liu et al., 2016).

Cotton (*Gossypium* spp.) is one of the most economically important crop plants and is the most important textile fibre crop in the world. Verticillium wilt, caused by the soil-borne fungal pathogen *Verticillium dahliae*, is the most destructive cotton disease worldwide. *Gossypium hirsutum* and *Gossypium barbadense* are the most widely cultivated cotton species today and originate from the interspecific hybridization between the A-genome species *Gossypium arboreum* (A2) and the D-genome species *Gossypium raimondii* (D5). *G. hirsutum* has a higher yield but a weaker Verticillium wilt resistance, while *G. barbadense* has a lower yield but a higher resistance to Verticillium wilt (Wang et al., 2008; Wang et al., 2012). Chromosome segment substitution lines (CSSLs), known as interspecific introgression lines, possess the same genetic background as the recurrent parent. Recently, cotton CSSLs have been developed by transferring valuable alien genes from *G*. *barbadense* to improve the *G. hirsutum* species (Saha et al., 2013; Li et al., 2016; Zhai et al., 2016). However, a systematic analysis of cotton circRNAs has not been reported thus far. In this study, two CSSLs of *G. barbadense* introgressed into *G. hirsutum*, a line highly resistant to Verticillium wilt (named CSSL-1) and a line susceptible to Verticillium wilt (named CSSL-4), were used to study the role of circRNAs in the cotton Verticillium wilt response through an RNA-seq approach. Our results will enrich the knowledge on plant circRNAs and help to reveal the role and mechanism of circRNAs in cotton Verticillium wilt resistance.

## Materials and Methods

### Materials and growth conditions

A highly aggressive defoliating fungus, *Verticillium dahliae* strain V991 (Sun et al., 2013; Zhang et al., 2012), was used for inoculation. For conidial production, V991 was cultured on potato dextrose agar (PDA) and was then inoculated into liquid Czapek’s medium at 25 °C for 7 days. The concentration of the conidial suspensions was adjusted to 1 ×10^7^ conidia/mL with sterile distilled water for subsequent use.

Two CSSLs of *G.   barbadense* introgressed in *G. hirsutum*, CSSL-1 and CSSL-4, derived from an interspecific cross of *G. hirsutum* (CCRI36) ×*G. barbadense* (Hai1), in which CCRI36 is a susceptible cultivar (*G. hirsutum* L.) and Hai1 is a highly resistant line (*G. barbadense* L.), were obtained from the Cotton Institute of the Chinese Academy of Agricultural Sciences of China. CSSL-1 is highly resistant to Verticillium wilt, while CSSL-4 is susceptible to Verticillium wilt (Shi et al., 2016).

CSSL-1 and CSSL-4 were cultivated in a controlled-environment chamber under a 16 h light/8 h dark photoperiod with 80% relative humidity at 28/25 °C (day/night). The roots of CSSL-1 and CSSL-4 in the two-true-leaf seedling stage were inoculated with a *V. dahliae* conidia suspension using a root dip method (He et al., 2014). Control plants were mock-inoculated with sterile water. The roots and stems were harvested at the squaring stage in the adult period after inoculation and included the following eight samples: Root of the Inoculated Resistant line CSSL-1 (C-RRI); Stem of the Inoculated Resistant line CSSL-1 (C-SRI); Root of the Inoculated Susceptible line CSSL-4 (C-RSI); Stem of the Inoculated Susceptible line CSSL-4 (C-SSI); Root of the Mock Resistant line CSSL-1 (C-RRM); Stem of the Mock Resistant line CSSL-1 (C-SRM); Root of the Mock Susceptible line CSSL-4 (C-RSM); and Stem of the Mock Susceptible line CSSL-4 (C-SSM).

### Library preparation and Illumina sequencing

Total RNA was extracted from eight samples with three biological replicates to construct RNA-seq libraries. In total, 24 libraries were constructed: C-RRI-1, C-RRI-2, C-RRI-3, C-SRI-1, C-SRI-2, C-SRI-3, C-RSI-1, C-RSI-2, C-RSI-3, C-SSI-1, C-SSI-2, C-SSI-3, C-RRM-1, C-RRM-2, C-RRM-3, C-SRM-1, C-SRM-2, C-SRM-3, C-RSM-1, C-RSM-2, C-RSM-3, C-SSM-1, C-SSM-2, and C-SSM-3. The TRIzol reagent (Invitrogen) was used to extract the total RNA following the manufacturer’s instructions. The quality and concentration of the extracted total RNA was validated using an ultraviolet spectrometer and electrophoresis on a denaturing formaldehyde agarose gel. The Ribo-Zero Magnetic Kit (Epicentre, Madison, WI, USA) was used to remove the rRNAs, and RNase R (Sigma Aldrich, St. Louis, MO, USA) was added to digest the linear RNAs. The circRNAs were then disrupted into short fragments using fragmentation buffer (Ambion). Using these short fragments as templates, the first-strand cDNA was synthesized using random hexamer primers, and the second-strand cDNA was synthesized by adding buffer, RNase H, dNTPs, and DNA polymerase I. Then, the resulting short fragments were purified using a QiaQuick PCR (Qiagen, Valencia, CA, USA) purification kit and eluted using EB buffer for end repair, and adenine (A) and sequencing adapters were added. Suitable fragments were collected through agarose gel electrophoresis and selected as templates for PCR to form the RNA-seq libraries. Finally, the libraries were sequenced using an Illumina Hiseq 2000 sequencer.

### Read mapping and identification and classification of the circRNAs

The low-quality reads, adaptor reads, and rRNA reads were removed from the raw sequencing RNA-seq reads and were uniquely aligned using TopHat2 (Kim et al., 2013) against the *G. hirsutum* genome (Gossypium_Hirsutum_v1.1 version) downloaded from the Cotton Research Institute, Nanjing Agricultural University (http://mascotton.njau.edu.cn/). The reads that did not map to the reference genome were named Unmapped Reads, and 20 bp from the two ends of Unmapped Reads were extracted and named the Anchors Reads. The Anchors Reads were independently mapped to the reference genome, and find_circ (v1.2, https://github.com/marvin-jens/find_circ) was used with the default parameters to analyse and identify the candidate circRNAs. Novel circRNAs were identified by blast searches against the circBase database (http://www.circbase.org/) using the candidate circRNAs with *E*-value of <e^−10^.

A circRNA was formed between the junction of a downstream splice donor and an upstream splice acceptor. The end points of the downstream donor and the upstream acceptor were a pair of breakpoints. The circRNAs were further classified into six types according to the location of the pair of breakpoints and the circRNA’s origin in the genome as follows: annot_exons (the pair of breakpoints was located in the starting point of an exon and the end point of another exon and the circRNA originated from the multiple exons between the breakpoints); one_exon (the pair of breakpoints was located in a same exon and the circRNA originated from the nucleotides between the breakpoints); intronic (the pair of breakpoints was located in the same intron and the circRNA originated from the nucleotides between the breakpoints); exon_intron (the circRNA contained one or multiple exons, but the breakpoints were not located at the starting point of an exon or the end point of an exon. The circRNA originated from the nucleotides between the first breakpoint and the starting point of the first exon or multiple exons and the nucleotides between the end point of the last exon and the second breakpoint); intergenic (the pair of breakpoints was located in the fragment between two genes and the circRNA originated from the nucleotides between the breakpoints); and antisense (the circRNA originated from the antisense strand of a gene).

### Validation of circRNAs in cotton

Total RNA was isolated from root and stem of CSSL-1 and CSSL-4 seedlings using TRIzol reagent (Invitrogen, Carlsbad, CA, USA) according to the manufacturer’s protocol, and used subsequent cDNA preparation with random primers. The divergent primers were then designed on the flanking sequences of head-to-tail splicing sites of circRNAs. The divergent primers, that is, the forward primer being located downstream of the reverse primer when they are aligned to genomic sequence. Polymerase chain reactions (PCRs) were done using the divergent primers and cDNA templates. PCR products were then separated using agarose gel electrophoresis, and purified with gel extraction kit. Sanger sequencing were performed to further confirm the presence of the back-spliced junction sites.

### Identification and analysis of circRNA source genes

The genes producing circRNAs were called the parental genes or source genes of circRNAs. When the reads containing head-to-tail splicing sites of circRNAs were uniquely aligned to the genomic DNA sequence of annotated genes, the circRNAs originated from the annotated genes and the annotated genes were called the source genes of the circRNAs. Due to their origination from the fragment between two genes, the intergenic circRNAs had no source genes and are described as ‘NA’. The source genes of the circRNAs were then subjected to Gene Ontology (GO) and KEGG Ontology (KO) analyses, and the cut-off for judging significant enrichment was set to a corrected *P*-value ≤0.05.

### Expression analysis of the circRNAs

The RPM (reads per million mapping) was used to obtain the relative expression levels of the circRNAs. Then, differential expression analysis of the circRNAs was performed using edgeR (Robinson, McCarthy & Smyth, 2010) with the default parameters. The fold change of the RPM values greater than 2 (—log2 (FC)— >1) and *P* value lower than 0.05 (*P* < 0.05) were used as the threshold for detecting differentially expressed circRNA.

## Results

### RNA sequencing and identification of circRNAs in cotton

To analyse the role of circRNAs in Verticillium wilt response, the two CSSLs of *G. barbadense* introgressed in *G. hirsutum*, CSSL-1 and CSSL-4, were inoculated with *V. dahliae*, and the root and stem of the inoculated plants and the plants in the control group were harvested to construct RNA-seq libraries. In total, 24 RNA-seq libraries (eight samples, C-RRI, C-RSI, C-RRM, C-RSM, C-SRI, C-SSI, C-SRM, and C-SSM, with three biological replicates) were constructed and sequenced. A total of 2,285,685,898 raw reads were obtained. After the low-quality reads, adaptor reads, and rRNA reads were filtered out, a total of 2,270,853,468 clean reads were used to align against the *G. hirsutum* genome. In total, 450,211,774 reads (approximately 19.82%) did not map to the reference genome and were used to identify the circRNAs using find_circ. Based on the sequence reads, 686 circRNA candidates were identified and named from ghi_circ_000001 to ghi_circ_000686 (Table S1, Dataset S1). The identified circRNAs were all novel circRNAs based on the results of BLAST searches against the circBase database with an *E*-value of <e^−10^.

**Figure 1:**
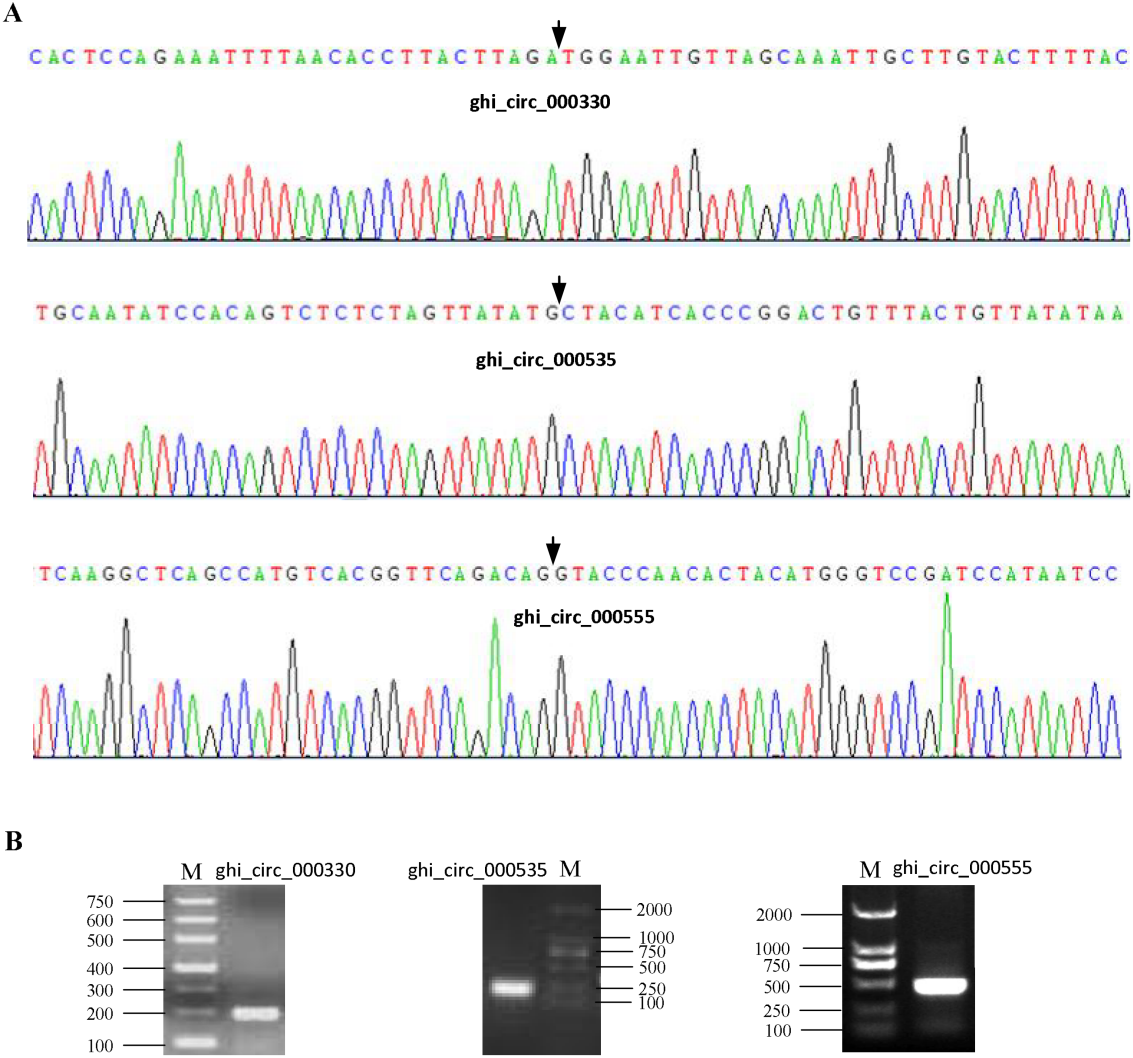
Experimental validation of circRNAs in cotton. (A) The results of Sanger sequencing. The black arrow represents the head-to-tail back-spliced site. (B) The results of agarose gel electrophoresis. The letter M represents DNA marker.

To confirm our identification circRNAs, divergent primers (Table S2) were designed for three randomly selected circRNAs to perform PCR. The PCR products were further analyzed by agarose gel electrophoresis and Sanger sequencing. The results showed that all circRNAs had bands of expected size and validated back-spliced junction sites (Fig. 1), indicating that our identification of circRNA are credible.

### Properties of cotton circRNAs

**Figure 2:**
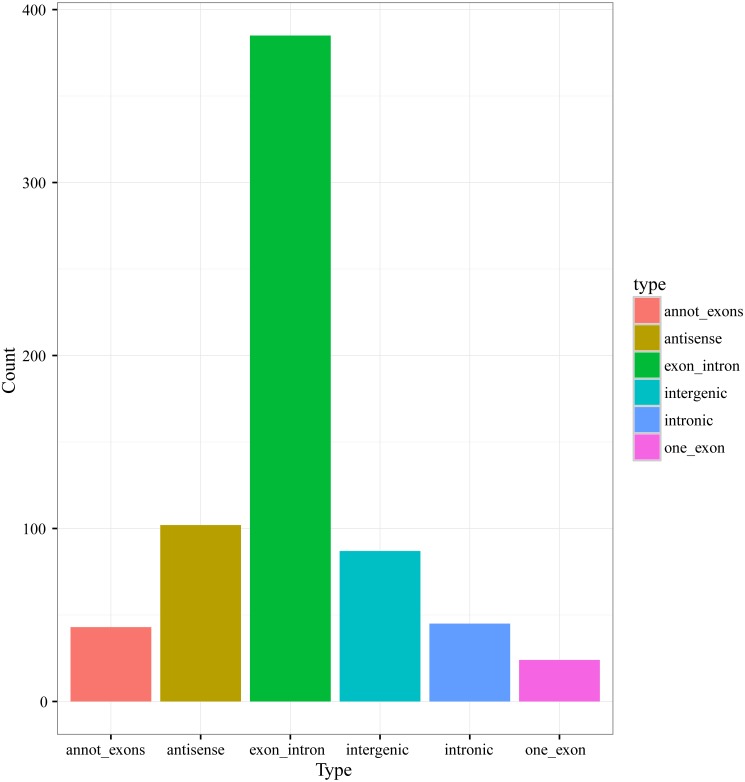
Numbers of circRNAs in each types.

According to the genome origination, the 686 circRNAs were classified into six types, including annot_exons, one_exon, intronic, exon_intron, intergenic, and antisense, containing 43, 24, 45, 385, 87, and 102 circRNAs, respectively (Fig. 2). The members of the exon_intron type were the most prevalent among the six types. The vast majority of the circRNAs, including the annot_exons, one_exon, intronic, and exon_intron-type circRNAs (497/686), originated from the sense strands of annotated protein-coding genes, indicating that the origination mechanism of circRNAs is closely related to the splicing mechanism of precursor mRNA (pre-mRNA), which is consistent with previous studies showing a non-canonical mode of RNA splicing or back-splicing events (Zhang et al., 2016; Salzman et al., 2012). The annot_exons, one_exon, and exon_intron-type circRNAs containing the exon sequence were in the majority of the 686 circRNAs (approximately 65.9%). The result is consistent with the studies in other species, such as *Arabidopsis thaliana* and *Oryza sativa*, whose circRNAs originated from exons of a single protein-coding gene (named Exonic circRNAs), accounting for 50.5% and 85.7%, respectively (Ye et al., 2015).

The length of circRNAs is uneven and ranges from approximately one hundred nucleotides to approximately 100 thousand nucleotides (Table S1). The mean length of the annot_exons, one_exon, intronic, exon_intron, intergenic, and antisense circRNAs are 530, 238, 30,878, 21,901, 14,719, and 19,326 nucleotides, respectively, indicating that the circRNAs containing exons (annot_exons, one_exon, and exon_intron) were shorter than the other circRNAs (intronic, intergenic, and antisense), and the circRNAs originating only from exons (annot_exons and one_exon) were very short, only several hundred nucleotides. These differences are due to having more and longer non-coding regions than the protein-coding regions in the eukaryotic genome.

**Figure 3:**
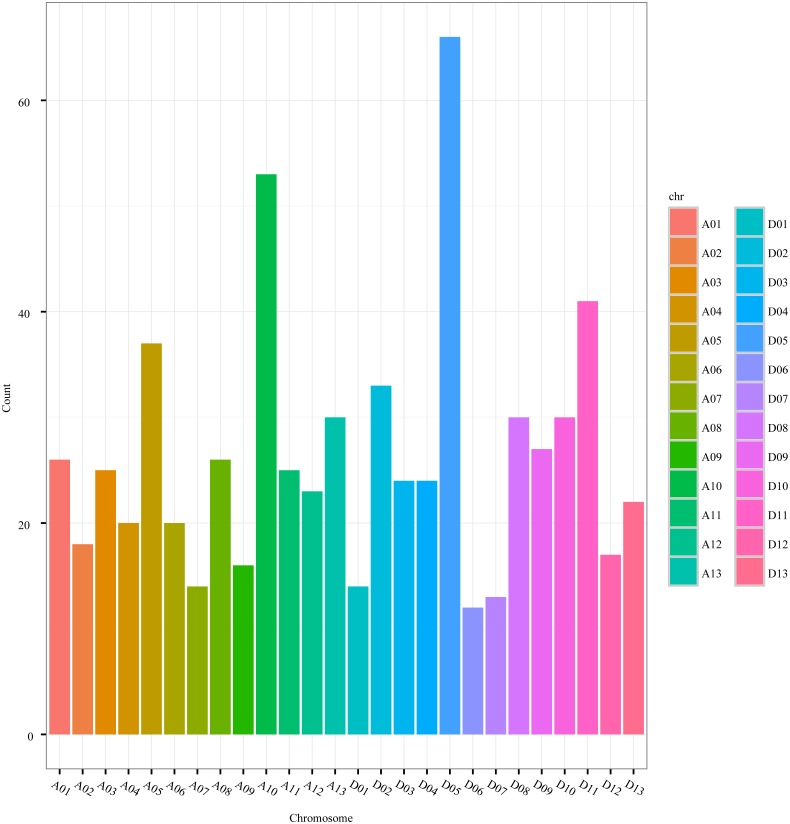
The circRNA distribution on *G. hirsutum* chromosomes. A01–A13 represent A-genome chromosomes 01–13, respectively, while D01–D13 represent D-genome chromosomes 01–13, respectively.

The distribution of circRNAs was also uneven among the chromosomes. The number of circRNAs on the A-genome chromosomes (A01-A13) of *G. hirsutum* ranged from 14 to 53, while the number on the D-genome chromosomes (D01-D13) ranged from 12 to 66 (Fig. 3, Table S1). Nevertheless, the distribution of the circRNAs was even between the A-genome and D-genome of *G. hirsutum*, 333 and 332 circRNAs, respectively, indicating that the contributions of the two subgenomes to generating the circRNAs were approximately equal. Thus, circRNAs are important and universal in cotton as in other species, including archaea, mammals, and other plants (Danan et al., 2012; Salzman et al., 2012; Jiang et al., 2014; Gruner et al., 2016; Lu et al., 2015; Ye et al., 2015; Sun et al., 2016; Darbani, Noeparvar & Borg, 2016).

### Identification of circRNA source genes

The annotated genes producing circRNAs were the source genes of the circRNAs, while the circRNAs without source genes were described as ‘NA’. A total of 599 of the identified 686 circRNAs originated from 416 source genes, and 87 intergenic-type circRNAs originated from the fragment between two genes, having no source genes (Table S1). More commonly, a source gene generated a single circRNA, but 103 genes generated two or more circRNAs, totalling 286 circRNAs. For example, the Gh_A05G3459 and Gh_D05G1184 genes each generated seven and eight circRNAs, respectively (Table S1). The result was also consistent with the conclusion that circRNAs possess an alternative splicing pattern (Zhang et al., 2016; Gao et al., 2016).

### Functional categorization of circRNA source genes

**Figure 4:**
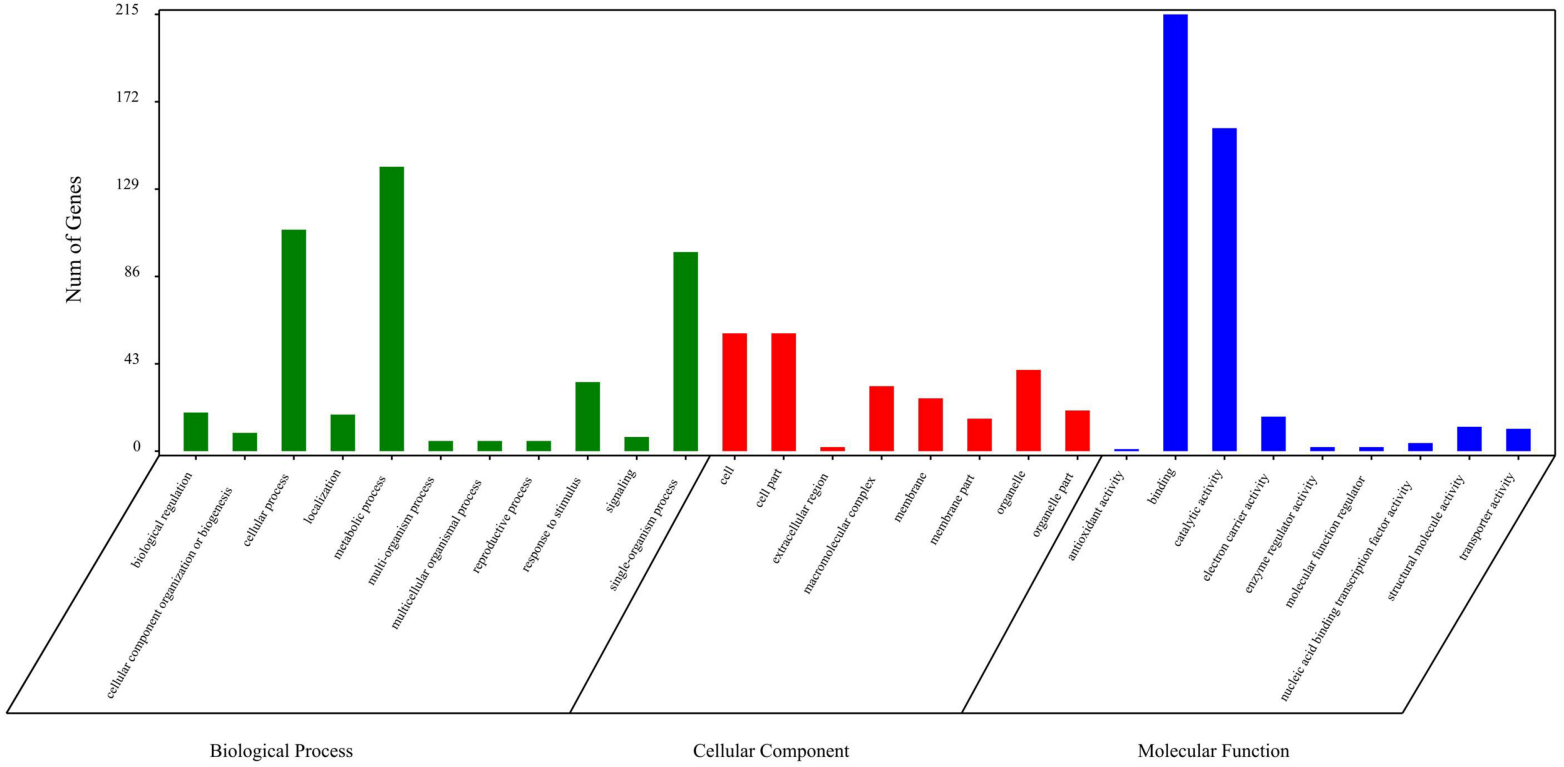
Gene Ontology classification of circRNA source genes.

**Figure 5:**
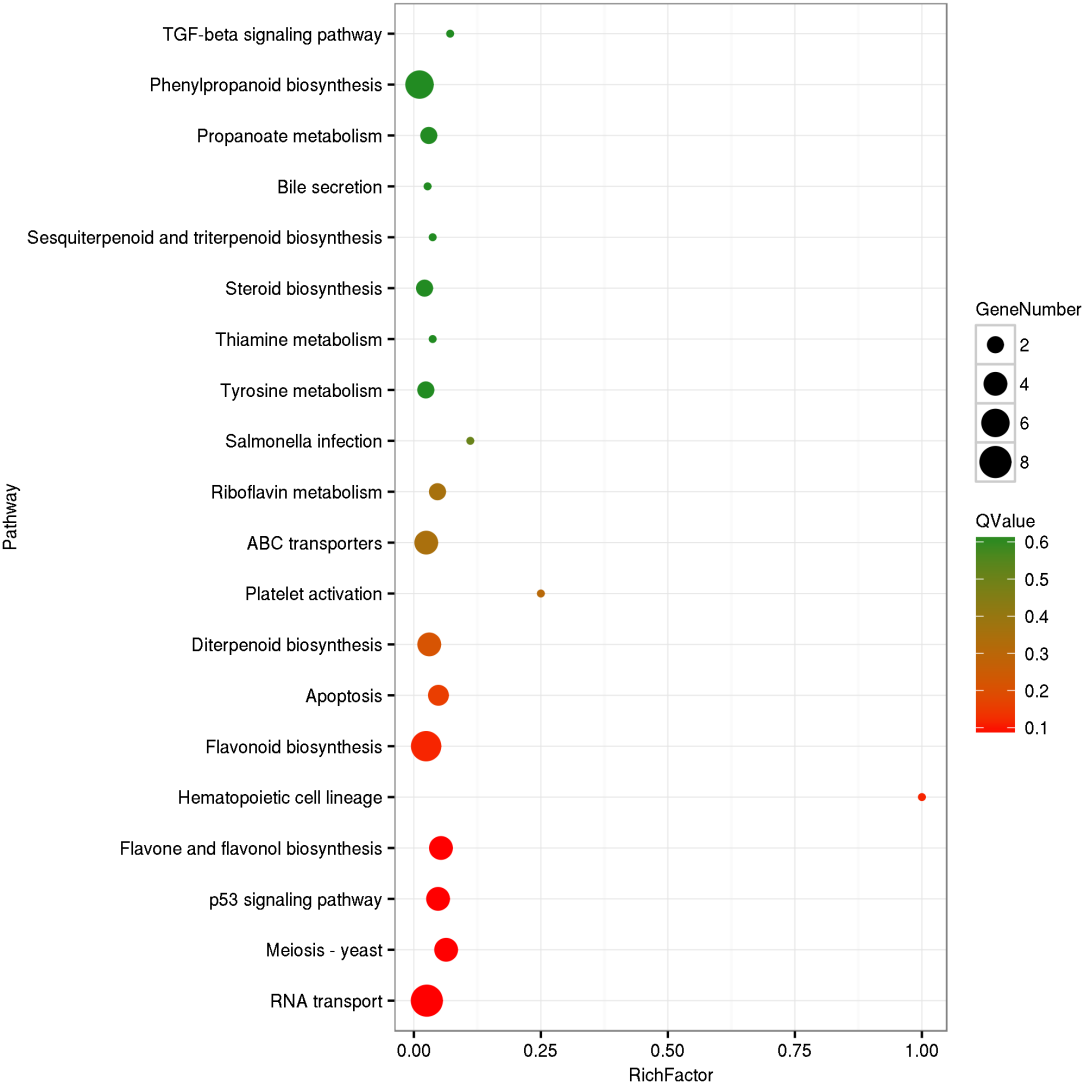
KEGG Ontology classification of circRNA source genes. The top 20 of pathway enrichment were shown. Rich Factor is the ratio between number of circRNA source genes and that of *G. hirsutum* genes in a pathway enrichment.

The circRNA source genes were subjected to GO and KO analyses. The GO categories indicated the functions of the source genes. The GO contains three main functional categories, biological process, cellular component, and molecular function. The 416 source genes were further categorized into 11 biological process terms, eight cellular component terms, and nine molecular function terms, in total 28 functional terms (Fig. 4, Table S3). The major enrichments for the biological process terms were ‘cellular process’, ‘metabolic process’, ‘single-organism process’, and ‘response to stimulus’. The 34 source genes in the ‘stimulus response’ term may function in response to Verticillium wilt. Further analysis showed that 20 of the 34 genes belong to the NBS (nucleotide binding site) gene family (Table S4), which is the largest disease-resistance gene family in plants and plays a central role in recognizing effectors from pathogens and in triggering downstream signalling during a plant’s response to pathogen invasion (Yue et al., 2012; McHale et al., 2006). The major enrichments for molecular function terms were ‘binding’ and ‘catalytic activity’. The levels of enrichment were not obviously different among the cellular component terms except that the ‘extracellular region’ term contained significantly fewer genes. The KO categories were used to reflect the biology pathway of the source genes, and 251 of the 416 the source genes were assigned to 91 KEGG pathways (Table S5). The top 20 pathway enrichments are shown in Fig. 5. The major pathways were ‘RNA transport’, ‘flavonoid biosynthesis’, ‘phenylpropanoid biosynthesis’, and ‘ribosome’, containing eight, seven, six and six genes, respectively.

### The relative expression analysis of circRNAs in tissues of cotton under different treatments

**Figure 6:**
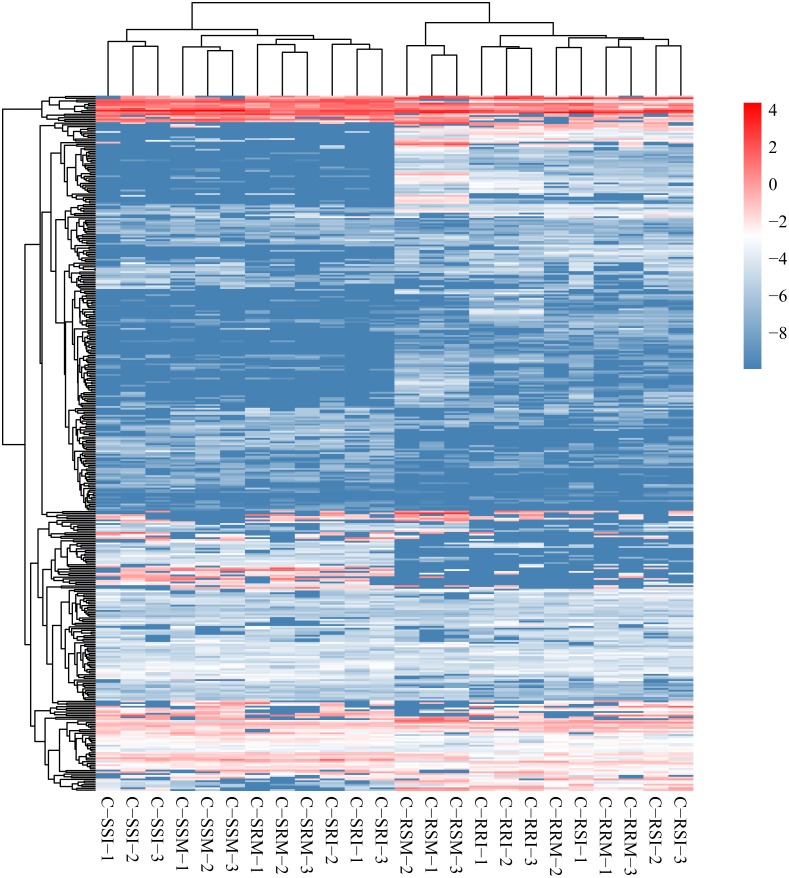
Heat map and clustering of circRNA expression profiles in different samples. Expression level is computed using log2 (expression quantity). Red color indicates higher level of the circRNA expression and green color represents lower level.

The relative expression of the identified 686 circRNAs was analysed among the eight root and stem samples of inoculated and mock CSSL-1 and CSSL-4, C-RRI, C-RSI, C-RRM, C-RSM, C-SRI, C-SSI, C-SRM, and C-SSM. The relative expression levels of the circRNAs in each library (Fig. 6, Table S6) were obtained according to the RPM method. The mean RPM value of a circRNA from three biological replicates was used as the relative expression level of the circRNAs of the samples (Table S7) for further analysis.

**Figure 7:**
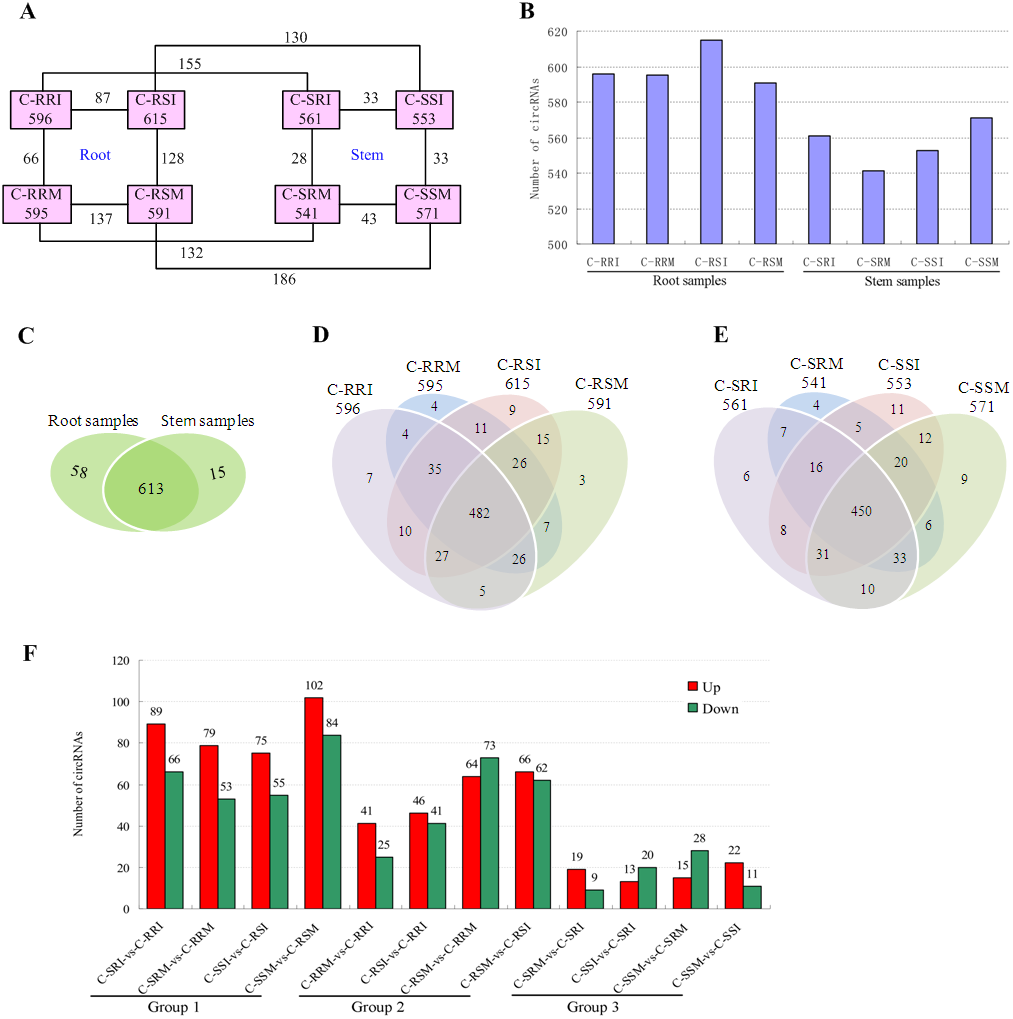
Quantity statistics of circRNAs existing in each sample and differentially expressed in each comparison. (A) Number of circRNAs existing in each sample and differentially expressed in each comparison. Pink boxes represented the eight samples, C-RRI, C-RRM, C-RSI, C-RSM, C-SRI, C-SRM, C-SSI, and C-SSM, and the number of circRNAs existing in each sample was written in the pink boxes; the numbers near the black lines linking two pink boxes are the numbers of circRNAs differentially expressed between the two samples. (B) Column chart of circRNAs detected in each sample. (C) Venn diagrams of circRNAs detected in root samples and stem samples. (D) Venn diagrams of circRNAs detected in each root sample. (E) Venn diagrams of circRNAs detected in each stem sample. (F) Column chart of circRNAs differentially expressed in each comparison. The numbers on column showed quantity of up-regulated (red) and down-regulated (green) circRNAs. The results of 12 comparisons are shown. C-SRI vs C-RRI, C-SRM vs C-RRM, C-SSI vs C-RSI, and C-SSM vs C-RSM, the comparisons between stem and root samples, were divided to Group 1; C-RRM vs C-RRI, C-RSI vs C-RRI, C-RSM vs C-RRM, and C-RSM vs C-RSI, the comparisons between two root samples, were divided to Group 2; C-SRM vs C-SRI, C-SSI vs C-SRI, C-SSM vs C-SRM, and C-SSM vs C-SSI, the comparisons between two stem samples, were divided to Group 3.

**Figure 8:**
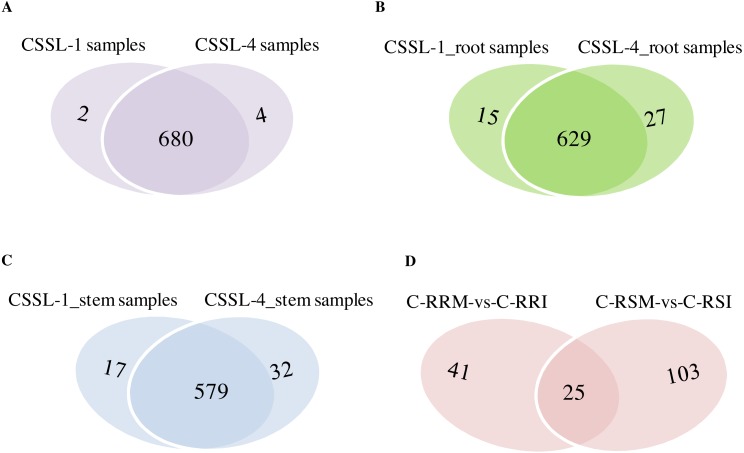
Comparison of circRNAs between CSSL-1 and CSSL-4. (A) Venn diagrams of circRNAs detected in CSSL-1 samples (C-RRI, C-RRM, C-SRI, and C-SRM) and CSSL-4 samples (C-RSI, C-RSM, C-SSI, and C-SSM). (B) Venn diagrams of circRNAs detected in Root_CSSL-1 samples (C-RRI and C-RRM) and Root_CSSL-4 samples (C-RSI and C-RSM). (C) Venn diagrams of circRNAs detected in Stem_CSSL-1 samples (C-SRI and C-SRM) and Stem_CSSL-4 samples (C-SSI and C-SSM). (D) Venn diagrams of circRNAs differentially expressed in two comparisons, C-RRM vs C-RRI and C-RSM vs C-RSI.

The number of circRNAs existing in each sample was shown in Fig. 7A. Most of the 686 circRNAs were detected in each sample (Fig. 7B, Table S7), and 319 of the 686 circRNAs were expressed in all 8 samples (Table S8). The CSSL-1 samples (C-RRI, C-RRM, C-SRI, and C-SRM) and CSSL-4 samples (C-RSI, C-RSM, C-SSI, and C-SSM) contained 680 common circRNAs and only 2 and 4 different circRNAs, respectively (Fig. 8A). Separately comparing the root and stem samples of CSSL-1 and CSSL-4, there were 629 and 579 common circRNAs and only 15 and 27, and 17 and 32 different circRNAs, respectively (Figs. 8B, 8C). These data indicated that the number of circRNAs detected in the CSSL-1 and CSSL-4 samples was not different. The distribution of the circRNAs among the root samples showed that 482 circRNAs (70.3%) were detected in all four samples, and 663 (96.6%) were detected in at least two of the four samples (Fig. 7D). The distributions of the circRNAs among stem samples showed that 450 circRNAs (65.6%) were detected in all four samples, and 656 (95.6%) were detected in at least two of the four samples (Fig. 7E). These results indicated that circRNAs were widespread in tissues. However, the root samples (C-RRI, C-RRM, C-RSI, and C-RSM) obviously contained more circRNAs than the stem samples (C-SRI, C-SRM, C-SSI, and C-SSM) (Fig. 7B). In addition, 15 of the 686 circRNAs were not detected in any of the root samples, while 58 of the 686 circRNAs were not detected in any of the stem samples (Fig. 7C), indicating that more circRNAs existed in the root of cotton than in the stem.

### CircRNA expression variations among the different samples

Variations in circRNA expression were identified based on 12 comparisons of C-SRI vs C-RRI, C-SRM vs C-RRM, C-SSI vs C-RSI, C-SSM vs C-RSM, C-RRM vs C-RRI, C-RSI vs C-RRI, C-RSM vs C-RRM, C-RSM vs C-RSI, C-SRM vs C-SRI, C-SSI vs C-SRI, C-SSM vs C-SRM, and C-SSM vs C-SSI. Differentially expressed circRNAs in the comparisons were detected according to —log2 (FC)— >1 and *P* < 0.05 (Figs. 7A, 7F, Tables S9– S20) to study their role in cotton Verticillium wilt resistance. The 12 comparisons were divided to three groups: Group 1 contained four comparisons between stem and root (C-SRI vs C-RRI, C-SRM vs C-RRM, C-SSI vs C-RSI, and C-SSM vs C-RSM), Group 2 contained four comparisons between two root samples (C-RRM vs C-RRI, C-RSI vs C-RRI, C-RSM vs C-RRM, and C-RSM vs C-RSI), and Group 3 contained four comparisons between two stem samples (C-SRM vs C-SRI, C-SSI vs C-SRI, C-SSM vs C-SRM, and C-SSM vs C-SSI) (Fig. 7F).

The results showed that 391 of the 686 circRNAs were differentially expressed in at least one of the 12 comparisons (Table S21). A total of 280 of the 686 circRNAs were differentially expressed in at least one of the eight comparisons in Group 2 and Group 3 (Table S21).

The number of differentially expressed circRNAs in the comparisons in Group 1 was generally greater than that in the comparisons in Group 2 and Group 3. Additionally, each comparison in Group 1 exhibited more up-regulated circRNAs than down-regulated circRNAs (Fig. 7F), which was related to the above results showing that the number of circRNAs expressed in the roots was obviously greater than that in the stems. The results indicated that different circRNAs are expressed in different tissues, which is consistent with the characteristic that circRNAs often show tissue-, cell- or developmental stage-specific expression (Sun et al., 2016; Memczak et al., 2013; Salzman et al., 2013). Therefore, *G. hirsutum* should have non-identified and novel circRNAs in other tissues, and the 280 circRNAs differentially expressed in at least one of the 8 comparisons between two root or stem samples may be related to the cotton Verticillium wilt response. Moreover, the number of circRNAs that were differentially expressed in the root comparisons in Group 2 was much higher than that in the stem comparisons in Group 3 (Fig. 7F), indicating that the circRNAs exhibited a stronger and more rapid response to *V. dahliae* in the root. Therefore, the differentially expressed circRNAs in root comparisons were further analysed.

**Figure 9:**
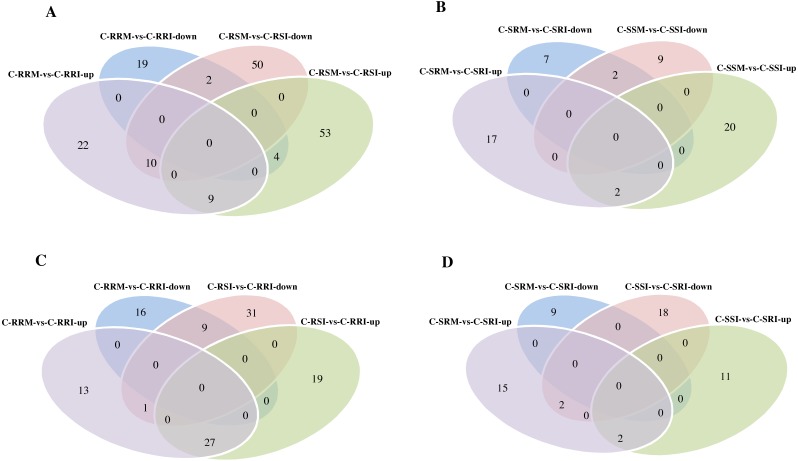
Comparison of differential expressed circRNAs being divided into up-regulated circRNAs and down-regulated circRNAs between two sample comparisons. (A) Venn diagrams of circRNAs differentially expressed in two root comparisons, C-RRM vs C-RRI and C-RSM vs C-RSI. (B) Venn diagrams of circRNAs differentially expressed in two stem comparisons, C-SRM vs C-SRI and C-SSM vs C-SSI. (C) Venn diagrams of circRNAs differentially expressed in two root comparisons, C-RRM vs C-RRI and C-RSI vs C-RRI. (D) Venn diagrams of circRNAs differentially expressed in two stem comparisons, C-SRM vs C-SRI and C-SSI vs C-SRI.

**Figure 10:**
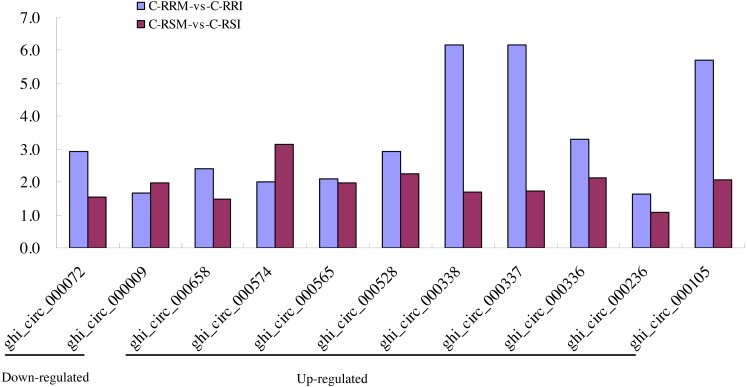
Comparison of the differential expression level of 9 common up-regulated and 2 common down-regulated circRNAs between C-RRM-vs-C-RRI and C-RSM-vs-C-RSI.

Firstly, the differentially expressed circRNAs in CSSL-1 and CSSL-4 root comparisons, C-RRM-vs-C-RRI and C-RSM-vs-C-RSI, were analysed and compared. The number of circRNAs differentially expressed in C-RRM-vs-C-RRI in the inoculated CSSL-1 was only approximately half of that in C-RSM-vs-C-RSI in the inoculated CSSL-4 (Fig. 8D). More differentially expressed circRNAs in the susceptible line compared to the resistant line indicated that the differential expression of circRNAs may play a role in the cotton Verticillium wilt response. There were 25 differentially expressed circRNAs in common in C-RRM-vs-C-RRI and C-RSM-vs-C-RSI (Fig. 8D). In addition, nine and two of the 25 circRNAs were up-regulated and down-regulated, respectively, in both C-RRM-vs-C-RRI and C-RSM-vs-C-RSI (Fig. 9A), and the differential expression level analysis of the total 11 common circRNAs showed that their levels were generally higher in C-RRM-vs-C-RRI than in C-RSM-vs-C-RSI (Fig. 10), indicating that the differential expression level might be one of the reasons for the resistance to Verticillium wilt differences in cotton. Therefore, the differentially expressed circRNAs may play a role in the resistance or susceptibility to Verticillium wilt.

Then, the differentially expressed circRNAs in the two C-RRI comparisons, C-RRI-vs-C-RRM and C-RRI-vs-C-RSI, were analysed and compared. There were 27 common up-regulated and nine common down-regulated circRNAs, for a total of 36 circRNAs, between the two C-RRI comparisons (Fig. 9C). These common circRNAs accounted for up to 54.5% and 41.4% of the total differentially expressed circRNAs in C-RRM-vs-C-RRI and C-RSI-vs-C-RRI, respectively. C-RRI is an inoculated resistant line sample, and its two comparisons (vs RRM, a mock resistant line sample, and vs C-RSI, an inoculated susceptible line sample) had a large proportion of common circRNAs, approximately 50%, which also indicated that the differentially expressed circRNAs may play a role in the response to Verticillium wilt.

Finally, the differentially expressed circRNAs in all the root comparisons were analysed. There were 12 common differentially expressed circRNAs in the four root comparisons (Table 1), speculating that the 12 circRNAs may be more sensitive to Verticillium wilt. Eleven of the 12 circRNAs had source genes (Table 1), and nine of the 11 source genes had defined functions according to the gene annotations of the *G. hirsutum* genome (Table 1). Notably, two source genes might respond to pathogen invasions. The Gh_A01G0389 gene, the source gene of ghi_circ_000009, was annotated as a D-mannose binding lectin protein gene with an apple-like carbohydrate-binding domain, which functions to specifically recognize diverse carbohydrates and mediate a wide variety of biological processes, such as cell–cell and host-pathogen interactions, serum glycoprotein turnover, and innate immune responses. The Gh_D01G0335 gene, source gene of ghi_circ_000336, was annotated as the NB-ARC domain-containing disease resistance protein gene belonging to the NBS gene family.

**Table 1:** The source genes and their annotation of the 12 circRNAs differentially expressed in all root comparisons.

CircRNA id	Source gene	Gene annotation
ghi_circ_000009	Gh_A01G0389	D-mannose binding lectin protein with Apple-like carbohydrate-binding domain
ghi_circ_000023	Gh_A01G1834	Sulfotransferase 16
ghi_circ_000034	Gh_A02G0219	Cytochrome P450
ghi_circ_000072	Gh_A04G0169	NA
ghi_circ_000115	Gh_A05G3458	Retinoblastoma-related 1
ghi_circ_000140	Gh_A06G1591	DNAJ heat shock N-terminal domain-containing protein
ghi_circ_000292	Gh_A12G0465	NA
ghi_circ_000309	NA	NA
ghi_circ_000328	Gh_A13G1798	2-oxoglutarate (2OG) and Fe(II)-dependent oxygenase superfamily protein
ghi_circ_000336	Gh_D01G0335	NB-ARC domain-containing disease resistance protein
ghi_circ_000468	Gh_D05G2355	Laccase 14
ghi_circ_000501	Gh_D06G0902	Glycosyl hydrolases family 32 protein

Considering the stem samples, the number of differentially expressed circRNAs in C-SSM-vs-C-SSI in the susceptible line was also greater than that in C-SRM-vs-C-SRI in the resistant line, but the difference was small (Fig. 7A). However, there were only four and two common differentially expressed circRNAs between C-SRM-vs-C-SRI and C-SSM-vs-C-SSI, and between C-SRM-vs-C-SRI and C-SSI-vs-C-SRI, respectively (Figs. 9B, 9D). Moreover, an expression analysis of the circRNAs in the stem comparisons showed that no circRNAs were differentially expressed in all four stem comparisons (Table S21). These results also indicated that the circRNAs exhibited a stronger, more rapid response to *V. dahliae* in the root than in the stem.

Above all, the differentially expressed circRNAs may play a role in Verticillium wilt resistance. A total of 280 circRNAs were differentially expressed in the root comparisons and stem comparisons in Group 2 and Group 3 (Table S21), and 247 of the 280 circRNAs had source genes (Table S22). According to the annotation of these source genes, 13 non-redundant source genes of the 17 circRNAs were related to defence response (GO:0006952) (Table 2, Table S22). A conserved domain analysis in NCBI showed that 11 of the 13 source genes were NBS family genes, containing an NB-ARC domain related to disease resistance (Table 2). Therefore, the NBS family of genes may play a role in Verticillium wilt resistance, and they might be regulated by circRNAs in the disease-resistance process in cotton.

**Table 2:** The circRNAs and their source genes related to defense response.

No.	CircRNA id	Source gene	Gene family
1	ghi_circ_000001	Gh_A01G0125	NBS gene family
2	ghi_circ_000274	Gh_A11G2684	NBS gene family
3	ghi_circ_000336	Gh_D01G0335	NBS gene family
4	ghi_circ_000337	Gh_D01G0335	NBS gene family
5	ghi_circ_000338	Gh_D01G0335	NBS gene family
6	ghi_circ_000353	Gh_D02G0093	NBS gene family
7	ghi_circ_000378	Gh_D02G1447	Pathogenesis-related protein Bet v I family
8	ghi_circ_000421	Gh_D04G1458	Pathogenesis-related protein Bet v I family
9	ghi_circ_000474	Gh_D05G3257	NBS gene family
10	ghi_circ_000475	Gh_D05G3336	NBS gene family
11	ghi_circ_000597	Gh_D10G2263	NBS gene family
12	ghi_circ_000598	Gh_D10G2263	NBS gene family
13	ghi_circ_000601	Gh_D10G2325	NBS gene family
14	ghi_circ_000606	Gh_D10G2422	NBS gene family
15	ghi_circ_000623	Gh_D11G2672	NBS gene family
16	ghi_circ_000635	Gh_D11G3108	NBS gene family
17	ghi_circ_000636	Gh_D11G3108	NBS gene family

## Discussion

Recently, studies showed that circRNAs exist in plants and play a role in responding to environmental stress (Ye et al., 2015; Zuo et al., 2016; Wang et al., 2017; Liu et al., 2016). Verticillium wilt is the most destructive disease in cotton production worldwide. To elucidate the role of circRNAs in cotton Verticillium wilt response, two CSSLs of *G. barbadense* introgressed in *G. hirsutum*, CSSL-1 (a highly resistant line) and CSSL-4 (a susceptible line), were used to construct an RNA-seq library and to analyse circRNA based on *V. dahliae* inoculation.

In contrast with the number of mammalian studies, few comprehensive studies on circRNAs in plants have been conducted thus far. According to publicly available RNA-Seq data, 12,037, 6,012, and 854 circRNAs were identified in rice, *Arabidopsis*, and tomato, respectively (Lu et al., 2015; Ye et al., 2015; Zuo et al., 2016). Recently, 88 and 5,372 circRNAs were identified and isolated in wheat and soybean, respectively (Wang et al., 2017; Zhao et al., 2017). Our identification and analysis of cotton circRNAs thus enriched plant circRNA research. Here, 686 novel circRNAs in cotton were identified, and the contributions of the two subgenomes (A-genome and D-genome) of *G. hirsutum* to the generated circRNAs was approximately the same, indicating that circRNAs are universally present in cotton.

Concerning the characteristics of circRNAs, first, the origination of circRNAs’ mechanism of precursor mRNA (pre-mRNA) splicing and alternative splicing was considered (Zhang et al., 2016; Salzman et al., 2012). In this study, with the exception of intergenic- and antisense-type circRNAs, the other circRNAs (497/686) originated from the sense strand of annotated protein-coding genes, indicating that the origination of circRNAs is closely related to the splicing mechanism of precursor mRNA (pre-mRNA). Moreover, 103 circRNA source genes generated two or more circRNAs, indicating that the circRNAs possessed an alternative splicing pattern (Zhang et al., 2016; Gao et al., 2016). Second, the circRNAs act as miRNA sponges (Wei et al., 2017; Hansen et al., 2013; Ebert, Neilson & Sharp, 2007; Franco-Zorrilla et al., 2007). In this study, the majority of circRNAs (approximately 65.9%), including the annot_exons, one_exon, and exon_intron types of circRNAs, contained the exon sequence, which is consistent with the results in *Arabidopsis thaliana* and *Oryza sativa*, showing approximately 50.5% and 85.7% exonic circRNAs (Ye et al., 2015), respectively. Acting as miRNA sponges is one of main functions of circRNAs, while miRNAs originate mainly from introns and target numerous mRNAs via combining completely or partially with mRNAs (Kim & Nam, 2006). The miRNAs combine with circRNAs and mRNAs; thus, the fact that the circRNAs originated mainly from exon-like mRNAs is reasonable. A close relationship among circRNAs, miRNAs, and mRNAs has been reported, and they may have a corresponding core sequence, which is why miRNAs can combine with circRNAs or mRNAs. However, the origination mechanism of the intergenic and antisense circRNAs remains unknown. The antisense-type circRNAs can also function as miRNA sponges. For example, the mammalian circRNA CDR1as (ciRS-7), an antisense-type circRNA, possesses 74 miR-7 binding sites and acts as a repressor of miR-7 (Hansen et al., 2013). Third, the expression of circRNAs has tissue-specificity (Sun et al., 2016; Memczak et al., 2013). In this study, an expression analysis of the circRNAs in different tissues showed that the number of circRNAs expressed in the root was obviously greater than in the stem, and the number of differentially expressed circRNAs in comparisons between the stem and the root was generally greater than for the comparisons between two root samples or stem samples. Therefore, non-identified and novel circRNAs should be present in the other tissues of *G. hirsutum*.

Our analysis of circRNAs in cotton showed that the differentially expressed circRNAs may play a role in cotton Verticillium wilt response. The differential expression of circRNAs are reported in rice responding to Pi-starvation stress (Ye et al., 2015). In wheat, 62 differentially expressed circRNAs are considered to be involved in the dehydration responsive process (Wang et al., 2017), and 163 circRNAs are expressed in response to chilling stress in tomato (Zuo et al., 2016). Verticillium wilt is the most destructive disease in cotton production. The study of circRNAs of two chromosome segment substitution lines, CSSL-1 (a highly resistant line) and CSSL-4 (a susceptible line), in Verticillium wilt response showed that the numbers of circRNAs in CSSL-1 and CSSL-4 was not different, and there were many common circRNAs. However, the number of differentially expressed circRNAs in CSSL-4 was much greater than that in CSSL-1 in response to *V. dahliae*. Meanwhile, the differential expression level of the common circRNAs in CSSL-1 was generally higher than that in CSSL-4. Moreover, C-RRI, as an inoculated resistant line treatment, and its two comparisons, C-RRM-vs-C-RRI and C-RSI-vs-C-RRI, had a large proportion (approximately half) of common differentially expressed circRNAs. These data indicated that the differentially expressed circRNAs may play a role in Verticillium wilt resistance in cotton.

Here, a total of 280 differentially expressed circRNAs were identified in cotton. The analysis of the source genes of these circRNAs showed that 13 non-redundant source genes of 17 differentially expressed circRNAs were related to a defence response, and 11 of the 13 source genes were NBS family genes (Table 2). GO analysis of the source genes of all 686 of the circRNAs showed that 20 of the 34 ‘stimulus response’ term genes belong to the NBS gene family (Table S4). Furthermore, 11, more than half of the 20 NBS genes, were the source genes of the differentially expressed circRNAs. Moreover, 12 circRNAs were commonly and differentially expressed in all the root comparisons (Table 1), and the source gene of ghi_circ_000336, the Gh_D01G0335 gene, was annotated as an NBS family gene. The NBS gene family is the largest disease resistance gene family in plants and plays a central role in recognizing effectors from pathogens and in triggering downstream signalling during the plant response to pathogen invasion (Yue et al., 2012; McHale et al., 2006; Zhao et al., 2017; Xiang et al., 2017). These results indicated that the NBS family genes in cotton may play a role in Verticillium wilt resistance and might be regulated by circRNAs in the disease-resistance process. Our results not only expanded the knowledge of circRNA in plant circRNAs, but also contributed to reveal the role and mechanism of circRNAs in the cotton Verticillium wilt response.

## Conclusions

A total of 686 novel circRNAs were identified in cotton. The circRNAs exhibit stronger and more rapid response to *V. dahliae* in root than in stem. A total of 280 differentially expressed circRNAs were identified and they may play a role in cotton Verticillium wilt response. The disease resistance-related genes, NBS family genes, may play a role in cotton Verticillium wilt resistance via being regulated by their circRNAs. Our study will help to reveal the role and mechanism of circRNAs in cotton Verticillium wilt resistance.

##  Supplemental Information

10.7717/peerj.4500/supp-1 Table S1 The origination informations of the identified circRNAs

10.7717/peerj.4500/supp-2 Table S2 The divergent primer sequences of circRNAs

10.7717/peerj.4500/supp-3 Table S3 GO classification of circRNA source genes

10.7717/peerj.4500/supp-4 Table S4 The stimulus response term gene annotation

10.7717/peerj.4500/supp-5 Table S5 KO classification of circRNA source genes

10.7717/peerj.4500/supp-6 Table S6 The circRNA expression in each library

10.7717/peerj.4500/supp-7 Table S7 The circRNA expression in each sample

10.7717/peerj.4500/supp-8 Table S8 The circRNA existing in all of the samples or only one of the samples

10.7717/peerj.4500/supp-9 Table S9 The differentially expressed circRNAs in comparison between C-SRI and C-RRI

10.7717/peerj.4500/supp-10 Table S10 The differentially expressed circRNAs in comparison between C-SRM and C-RRM

10.7717/peerj.4500/supp-11 Table S11 The differentially expressed circRNAs in comparison between C-SSI and C-RSI

10.7717/peerj.4500/supp-12 Table S12 The differentially expressed circRNAs in comparison between C-SSM and C-RSM

10.7717/peerj.4500/supp-13 Table S13 The differentially expressed circRNAs in comparison between C-RRM and C-RRI

10.7717/peerj.4500/supp-14 Table S14 The differentially expressed circRNAs in comparison between C-RSI and C-RRI

10.7717/peerj.4500/supp-15 Table S15 The differentially expressed circRNAs in comparison between C-RSM and C-RRM

10.7717/peerj.4500/supp-16 Table S16 The differentially expressed circRNAs in comparison between C-RSM and C-RSI

10.7717/peerj.4500/supp-17 Table S17 The differentially expressed circRNAs in comparison between C-SRM and C-SRI

10.7717/peerj.4500/supp-18 Table S18 The differentially expressed circRNAs in comparison between C-SSI and C-SRI

10.7717/peerj.4500/supp-19 Table S19 The differentially expressed circRNAs in comparison between C-SSM and C-SRM

10.7717/peerj.4500/supp-20 Table S20 The differentially expressed circRNAs in comparison between C-SSM and C-SSI

10.7717/peerj.4500/supp-21 Table S21 The differentially expressed circRNAs in 12 comparisons

10.7717/peerj.4500/supp-22 Table S22 The significantly differentially expressed circRNAs in each comparison

10.7717/peerj.4500/supp-23 Dataset S1 The nucleotide sequences of the 686 novel circRNAs identified from cotton

## Additional Information and Declarations

### Data Availability

The following information was supplied regarding data availability: The raw data is included in Dataset S1.
